# Trend Analysis of Substance Use Disorder During Pregnancy

**DOI:** 10.7759/cureus.23548

**Published:** 2022-03-27

**Authors:** Memory Ndanga, Saanie Sulley, Abimbola K Saka

**Affiliations:** 1 Health Information Management, Rutgers University, Piscataway, USA; 2 Health and Biomedical Informatics, Independent, Washington DC, USA; 3 Translational Research, Independent, Toronto, CAN

**Keywords:** drug addiction, socioeconomic, drug abuse, drug use, substance abuse, maternal drug use

## Abstract

Objectives: This study aims to analyze the trends in substance use among pregnant women in the United States.

Methodology: In this retrospective study, we utilized the National Inpatient Sample (NIS) dataset sponsored by the Agency for Healthcare Research and Quality (AHRQ) under the Healthcare Cost and Utilization Project (HCUP). Major Diagnostic Category (MDC) 14 (Pregnancy, Childbirth, and the Puerperium) and International Classification of Disease (ICD 10) codes were used to identify pregnancy-related diagnoses and presentations with any of the substance use disorder (SUD) indicators that met the inclusion criteria among the birthing population in the NIS dataset (2016-2018). We analyzed the demographic and regional characteristics between 2015 and 2018.

Results: Among the population, a total of 23,475 (2.7%) had a primary or secondary diagnosis of SUD, and 851,428 (97.3%) did not. In the study group of 332,275 (2.8%) that met the inclusion criteria, 12,750 (0.1%) use alcohol, 108,960 (0.9%) had opioid use disorder (OUD), 171,490 (1.4%) use cannabis, 6,375 (0.1%) use sedatives, 28,075 (0.2%) use cocaine, 48,765 (0.4%) use other stimulants, 1,155 (0%) use hallucinogens, 115 (0%) use inhalants, and 23,950 (0.2%) had other psychoactive diagnosis. Further analysis comparing the risk of severity and mortality at presentation, procedure type, delivery method, and cost of care shows statistically significant differences (p < 0.005) between the study and control groups.

Conclusion: The current trends necessitate a further assessment and implementation of comprehensive community-based treatment programs tailored to the most frequent regional SUD presentations, which could aid in mitigating drug use during pregnancy.

## Introduction

Substance abuse during pregnancy continues to increase in the United States, which has also seen an increase in the rate of women who experience severe health risks during pregnancy and subsequently an increase in the incidence of neonatal abstinence syndrome [1-5]. According to the federal Centers for Disease Control and Prevention (CDC), in 2019, 7% of US women reported that they had used prescription opioids during pregnancy, and one in five of those women reported misusing the drugs while pregnant [6]. It is estimated that about 5% of pregnant women use one or more addictive substances [7]. The type of drug being used and the degree of use, and the point of exposure all have an impact during pregnancy; therefore, it is essential to understand the trends of substance use in this population. A report by the Substance Abuse and Mental Health Services Administration (SAMHSA) also reflects that between 2015 and 2017, data on pregnant women and the use of illicit drugs, including cocaine, marijuana, and opioids, were alarming [8]. Opioid use is independently associated with increased odds of threatened preterm labor, early onset of delivery, poor fetal growth, and stillbirth [9], and opioid use was noted to have drastically increased between 2002 and 2014 [10]. The number of infants diagnosed with neonatal abstinence syndrome (NAS) increased from 3.4 to 5.8 per 1,000 hospital birth, while one facility reported more than 50 cases per every 1,000 births in 2017 [11]. Several studies have also seen an increase in marijuana use, which is linked to low birth weight, impulsivity, attention disorders, and other cognitive-behavioral issues [12-14]. A national survey conducted in the United States in 2012 showed that 15.9% of pregnant women respondents smoked cigarettes, 8.5% consumed alcohol, and 5.9% used illicit drugs [15]. Another study by researcher Kimberly Yonkers suggested that pregnant women's use of tobacco products and alcohol has also been noted to be almost 19%, a substantial problem that may be more prevalent than previously thought [16]. Alcohol was also associated with decreased head size from mid‐pregnancy to childhood and reduced development at 2.5 years [17]. Other drugs such as cocaine are also known to increase complications such as hypertension, myocardial infarction and ischemia, renal failure, hepatic rupture, and maternal death [18]. Drug use during pregnancy does not just affect the mother; therefore, there is a need to look at the trend in drug use during pregnancy to aid in identifying social determinants of health (SDOH).

## Materials and methods

In this retrospective study, we utilized the National Inpatient Sample (NIS) dataset sponsored by the Agency for Healthcare Research and Quality (AHRQ) under the Healthcare Cost and Utilization Project (HCUP). The NIS dataset is the largest all-payer dataset with a significant number of acute care inpatient hospitalizations in the United States. The NIS dataset provides access to clinical and service utilization of hospitalization discharge in a de-identified manner, protecting patients, hospitals, and physicians' privacy. Using Major Diagnostic Category (MDC) 14 (Pregnancy, Childbirth, and the Puerperium) and International Classification of Disease (ICD 10) codes, we identified pregnancy-related diagnoses and presentations with any of the substance use disorder (SUD) (F10.XXX, F11.XXX, F12.XXX, F13.XXX, F14.XXX, F15.XXX, F16.XXX, F18.XXX, and F19.XXX) indicators that met the inclusion criteria among the birthing population in the NIS dataset (2016-2018). We also utilized the World Health Organization (WHO) definition for women of reproductive age (15-49 years) as an inclusion criterion.

This study utilized IBM Statistical Package for Social Sciences (SPSS) version 23.0 (IBM Corp., Armonk, NY, USA). The analysis of the HCUP-NIS data from 2016 to 2018 adhered to HCUP analytic guidelines. Linear and logistic regressions were conducted to assess the effects and trends of selected variables on hospitalization outcomes among the pregnant population with any of the SUD codes and those without (control). Records with missing data were excluded from all calculations. Descriptive analysis was conducted to obtain the most frequent procedures among the selected population. Patient-level demographic data analyzed SUD and non-SUD diagnoses. Hospitalization outcome trends were analyzed using a linear and binary regression for selected variables. Cost of care was calculated using the total charge and the cost-to-charge ratio provided by the HCUP. The analysis was conducted through multiple linear and logistic regression models adjusted for the patient-level variables. The covariates included in the analysis include age group, year, income, insurance status, mortality and severity risk, region, patient location, number of procedures, and diagnosis. All p-values were two-tailed with a significance of p < 0.05.

## Results

**Figure 1:**
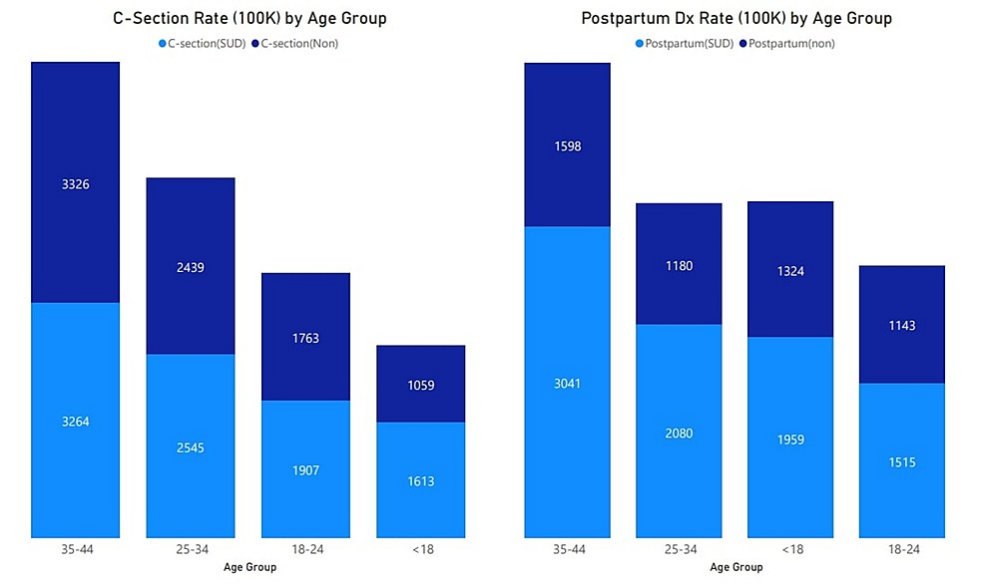
Rate of cesarean section and postpartum diagnoses per 100,000 pregnant patients by age group Comparison of cesarean section and postpartum diagnoses rate per 100,000 pregnant patients (2016–2018) among patients with (light blue) and without (dark blue) substance use disorder (SUD) by age group. A higher cesarean rate was observed among patients with SUD among age groups 25–34, 18–24, and less than 18. Higher postpartum diagnoses were also observed among pregnant patients with SUD (light blue) compared with those without (dark blue).

Among the population, a total of 23,475 (2.7%) had a primary or secondary diagnosis of SUD, and 851,428 (97.3%) did not, yielding 332,275 that met the inclusion criteria. The general characteristic of the population is shown in Figure 1. There were 12,750 (0.1%) alcohol users, 108,960 (0.9%) with opioid use disorder (OUD), 171,490 (1.4%) cannabis users, 6,375 (0.1%) sedative users, 28,075 (0.2%) cocaine users, 48,765 (0.4%) other stimulant users, 1,155 (0%) hallucinogen users, 115 (0%) inhalant users, and 23,950 (0.2%) with other psychoactive diagnosis. Further analysis comparing the risk of severity and mortality at presentation, procedure type, delivery method, and cost of care shows statistically significant differences (p < 0.005) between the study and control groups.

**Figure 2:**
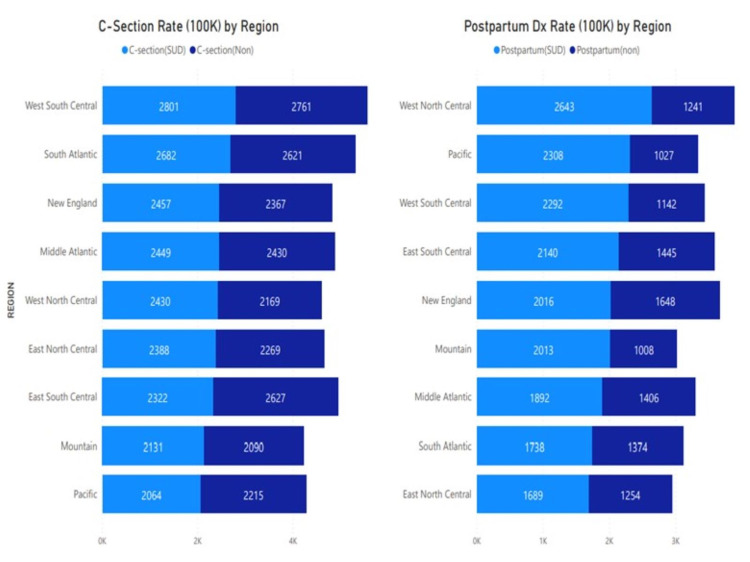
Rate of cesarean section and postpartum diagnoses per 100,000 pregnant patients by geographical regions Comparison of cesarean section and postpartum diagnoses rate per 100,000 pregnant patients (2016-2018) among patients with (light blue) and without (dark blue) substance use disorder (SUD) by geographic region. A higher rate of cesarean section was observed among pregnant patients with SUD in West South Central, South Atlantic, and New England regions. A significantly higher rate of postpartum diagnoses was observed among pregnant patients with SUD in the West North Central, Pacific, and West South-Central regions.

The number of diagnoses (NDX), cost of care, and length of stay (LOS) were all higher among the study group cesarean and non-cesarean deliveries. The cost of care for the population with SUD indicators was about $1,500 higher than the population without any SUD indicators. Hospitalizations with SUD indicators were more likely to have a higher LOS than those without as shown in Figure 2. Variance in LOS among patients with and without SUD indicators among postpartum diagnoses shows the significantly higher cost of care and number of diagnoses (NDX). Cost of care and NDX were higher among older populations for both study and control groups. Further stratification of severity and mortality risk at presentation by race and ethnicity shows a higher hospitalization rate among Native Americans, Black, and Hispanics. Moreover, NDX and LOS were significantly associated with a higher cost of care among all population groups.

Although length of stay (LOS) and NDX were similar among the study group by rural-urban classification, the cost of care was about $2,000 more for a patient in rural areas compared to urban areas. Areas with smaller population sizes showed a higher severity and cost at presentation among both groups. Cost of care was higher among the highest income quartile among both populations sets although variance in LOS among the groups was not significant. The payer observed significant variance in cost with the highest mean compensation Medicare ($6,014) and lowest self-pay ($4,541). The mortality rate among the study population was significantly higher at 18 compared to 4 among the control group. Native Americans and Blacks were more likely to have a higher NDX and LOS among both groups. LOS and NDX were not significantly different from one region to the next among the study population. Cost of care and racial and ethnic severity presentation varied across all regions of the United States. The highest mean cost of care for both groups was observed in the New England, Pacific, and Middle Atlantic regions.

**Figure 3:**
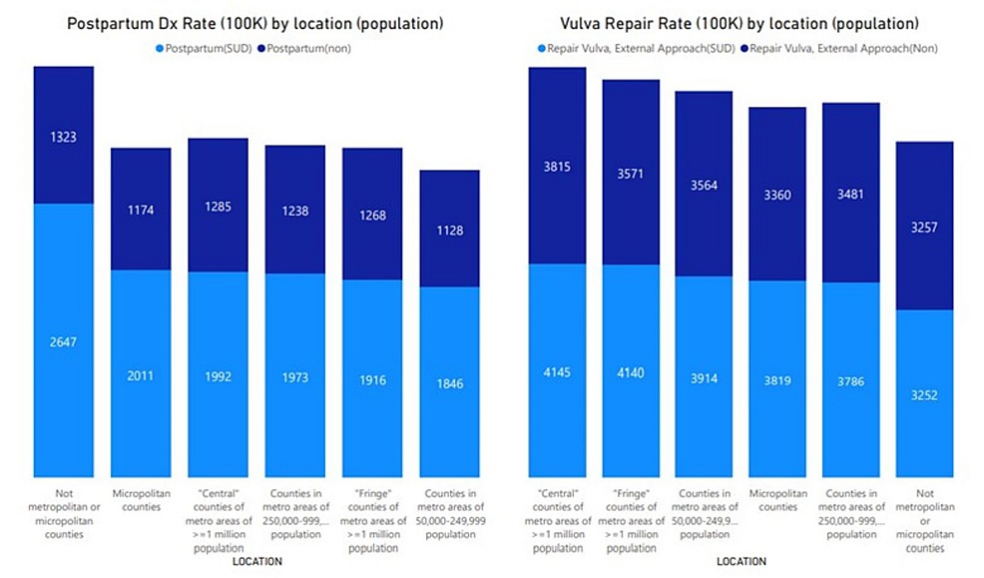
Rate of postpartum diagnoses and vulvar repair per 100,000 pregnant patients by population location Comparison of cesarean section and postpartum diagnoses rate per 100,000 pregnant patients (2016-2018) among patients with (light blue) and without (dark blue) substance use disorder (SUD) by location. The highest postpartum diagnoses were observed among populations with SUD in locations that were not metropolitan or micropolitan, followed by micropolitan and central counties with over one million population. The rate of vulvar repair was higher among pregnant patients with SUD in central counties with over one million population, followed by fringe counties and counties with populations less than 250,000.

Figure 3 shows SUD presentation by population sizes across the United States. The figure shows a higher SUD in smaller and rural communities. Patients in highly populated areas were more likely to have vulva repair compared to smaller populated areas.

**Figure 4:**
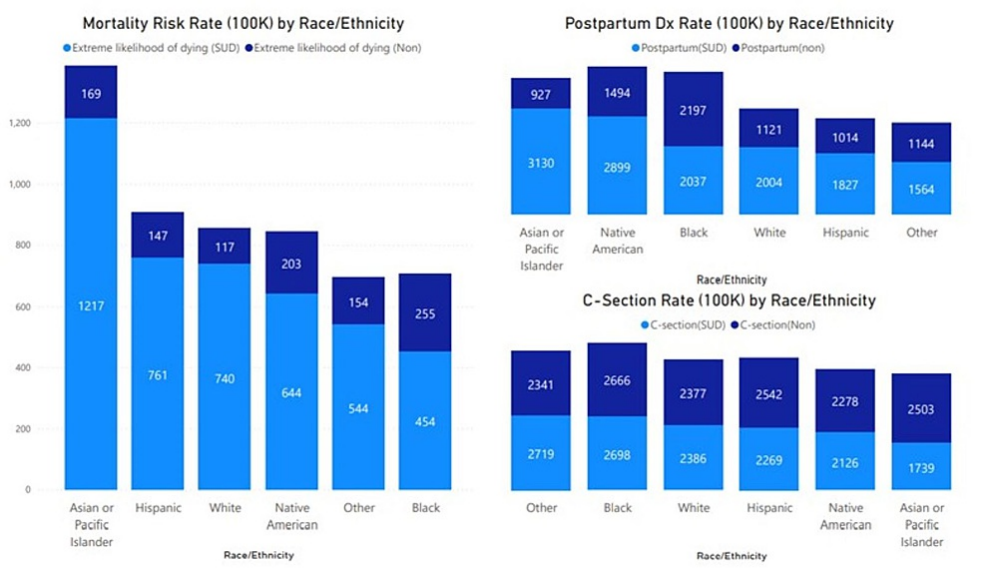
Rate of mortality risk, postpartum diagnoses, and cesarean section per 100,000 pregnant patients by race and ethnicity The highest mortality risk (extreme) at presentation among pregnant patients with SUD was observed among Asian or Pacific Islander, Hispanic, White, Native American, Other, and Black populations. Asian or Pacific Islander, Native American, Black, White, Hispanic, and Other pregnant patients with SUD were more likely to have a postpartum diagnosis. Cesarean section rate was the highest among pregnant patients with SUD among Other, Black, White, Hispanic, Native American, and Asian or Pacific Islander populations.

Figure 4 shows the variances presentation by race and ethnicity. The highest mortality risk at presentation was observed among the Asian/Pacific Islander, Hispanic, and White populations. The rate of cesarean section was higher among the Other, Black, and White population. Asian/Pacific Islanders, Native Americans, and Black were more likely to be diagnosed with postpartum complications.

## Discussion

This is the most recent study utilizing a national sample to access the diversity of factors associated with inpatient substance use and pregnancy in the United States. The utilization of cost of care instead of charges provides a better perspective of resource utilization. This study showed a prevalence of 2.7% SUD among the birthing population in the United States between 2016 and 2018. These presentations have been increasing over the years, especially among cannabis and opioid use disorders. The rate of presentation, as shown in this study, is about 1,205 per 100,000 births annually. High presentation rates were associated with socioeconomic and demographic indicators as shown by other studies [11,19].

Based on this study's findings, the resource utilization of patients was related to factors such as their geographic location and comorbidities. Although populations without SUD indicators had a more negligible cost and LOS, those in rural and micropolitan counties with limited care resources presented with higher severity risk, and their cost of care was higher. Studies have shown that initiatives focused on improving services and access to care for these populations using local dynamics are effective [20-22]. Although these initiatives have been developed to target these populations, it is imperative to evaluate other socioeconomic factors such as insurance or education, which may be preventing them from effectively accessing and participating in treatment programs.

The continuous trend of higher presentation among minority communities in all regions necessitates a reevaluation of current treatment and public health approaches. It is imperative to assess the factor contributing to the disproportionate presentation of a high severity among Native American and Black populations. It is also essential to access the factors related to the increasing trend of high LOS and costs among the Asian/Pacific Islander population. This study shows that SUD is not limited to low-income communities but is ubiquitous across communities in the United States. The lack of variance in the LOS and NDX among income demographics, as shown in this study, supports this conclusion. Therefore, this finding supports the diverse national, state, and regional efforts to address this issue from a public health perspective.

## Conclusions

The current trends necessitate further assessment and implementation of comprehensive community-based treatment programs tailored to the most frequent regional SUD presentations, which could aid in mitigating drug use during pregnancy. The higher severity at presentation in rural communities calls for more assessment of the drivers including socioeconomic opportunities, healthcare resource allocation, and other factors that could be contributing to these phenomena. The significantly higher cost of care, LOS, and NDX when comparing pregnant populations with and without any indicator of SUD shows the need for comprehensive plans to target this population. It also shows a need for educational programs in the prenatal period that addresses both long-term and short-term effects of SUD on pregnancy.

## Appendices

Table 1 shows logistic regression analysis conducted to assess the effects and trends of selected variables on hospitalization outcomes among the pregnant population.

**Table 1:** Logistic regression analysis for substance use among pregnancy childbirth presentation (2016-2018) CC: complication or comorbidity, MCC: major complication or comorbidity, LOS: length of stay, NDX: number of diagnoses, NPR, number of procedures

Variables	2016­–2018		P-value (Sig)
	OR	95% CI	P-value (Sig)
Extraction of products of conception, low, open approach	2.9	2.94–3.00	
Drainage of amniotic fluid, therapeutic from products of conception, via natural or artificial opening	1.03	1.02–1.04	<0.001
Ultrasonography	1.04	0.97–1.13	0.216
Extraction of products of conception, low, open approach	1.66	1.64–1.68	
Extraction of products of conception, vacuum, via natural or artificial opening	1.84	1.80–1.89	
Delivery of products of conception, external approach	2.02	2.00–2.04	
Repair perineum skin, external approach	1.80	1.78–1.83	<0.001
Dilation of the cervix, via natural or artificial opening	1.17	1.14–1.20	<0.001
Excision of bilateral fallopian tubes, open approach	0.83	0.85–0.88	<0.001
Repair vulva, external approach	0.86	0.84–0.88	<0.001
Division of female perineum, external approach	2.33	2.24–2.36	<0.001
Introduction of other hormones into a peripheral vein, percutaneous approach	1.13	1.12–1.14	<0.001
Introduction of hormone into female reproductive, via natural or artificial opening	1.02	1.00–1.04	0.024
Repair perineum muscle, open approach	3.15	3.10–3.20	<0.001
Other antepartum diagnoses without OR procedure with CC	9.40	9.07–9.74	
Other antepartum diagnoses without OR procedure with CC MCC	3.57	3.28–3.88	
Cesarean section without sterilization with CC	2.96	2.87–3.04	
Cesarean section with sterilization with CC	3.58	3.37–3.80	
Vaginal delivery without sterilization/d&c with cc	4.01	3.93–4.10	
Vaginal delivery with sterilization/d&c with mcc	2.93	2.22–3.85	
Vaginal delivery with sterilization/d&c with cc	5.27	4.72–5.89	
Postpartum and post-abortion diagnosis with OR procedure	1.25	1.18–1.32	
Postpartum and post-abortion diagnoses without OR procedure	1.64	1.60–1.68	
Age group (years)			
*<18			<0.001
18–24	6.66	6.48–6.85	<0.001
25–34	8.46	8.24–8.69	<0.001
35–44	6.00	5.85–6.16	<0.001
45–54	4.37	4.26–4.48	<0.001
Disposition			
Against medical advice	0.77	0.61–0.97	<0.001
Died	1.11	0.88–1.39	<0.001
Transfer to short-term hospital	3.38	2.68–4.26	0.467
Transfer other: includes skilled nursing facility	0.68	0.54–0.85	0.001
Home healthcare (HHC)	3.14	2.50–3.96	<0.001
*Routine			
LOS	0.97	0.97–0.98	0.91
NDX	1.23	1.23–1.24	<0.001
NPR	0.83	0.83–0.84	
Race			
*White			
Black	2.64	2.58–2.71	<0.001
Hispanic	1.56	1.52–1.60	
Asian or Pacific Islander	0.75	0.73–0.77	
Native American	0.45	0.43–0.47	
Other	3.72	3.58–3.86	
Census region			
*Northeast			
Middle Atlantic	0.99	0.97–1.01	0.80
East North Central	0.75	0.74–0.77	<0.001
West North Central	0.78	0.78–0.79	<0.001
South Atlantic	0.72	0.71–0.74	<0.001
East South Central	0.65	0.64–0.66	<0.001
West South Central	0.85	0.83–0.86	<0.001
Mountain	0.52	0.51–0.53	<0.001
Pacific	0.79	0.78–0.81	<0.001
Primary payer			
Medicare			
Medicaid	2.22	0.69–0.72	<0.001
Private insurance	3.01	0.99–1.02	
Self-pay	0.52	0.76–0.79	
No charge	2.91	1.12–1.16	
Other	2.85	1.26–1.34	
Rural-urban code			
*"Central" counties of metro areas of ≥1 million population			
"Fringe" counties of metro areas of ≥1 million population	0.90	0.22–0.91	<0.001
Counties in metro areas of 250,000–999,999 population	1.00	0.98–1.02	<0.657
Counties in metro areas of 50,000–249,999 population	1.06	1.04–1.08	<0.001
Micropolitan counties	1.15	1.13–1.18	<0.001
Not metropolitan or micropolitan counties	1.12	1.09–1.14	<0.001
Median household income			
*0–25th percentile			<0.001
26th–50th percentile (median)	1.73	1.73–1.71	
51st–75th percentile	1.44	1.4–1.46	
76th–100th percentile	1.25	1.23–1.27	
Severity of risk			
*No class specified			
Moderate loss of function	0.42	0.39–0.45	<0.001
Major loss of function	1.70	1.60–1.80	
Extreme loss of function	1.49	1.49–1.40	
Mortality risk (%)			
*No class specified			
Minor likelihood of dying	7.4	5.9–9.1	<0.001
Moderate likelihood of dying	2.0	1.8–2.2	
Major likelihood of dying	0.77	0.71–0.83	
Extreme likelihood of dying	0.69	0.64–0.75	
